
JOURNAL INFORMATION
==============================
NLM Title Abbreviation: ScientificWorldJournal
Journal ID: ScientificWorldJournal
NLM Journal ID: 101131163
Journal ID: TSWJ

The Scientific World Journal

ISSN: 2356-6140
EISSN: 1537-744X
Publisher: Wiley

ARTICLE INFORMATION
==============================
PMCID: PMC6009640
PMID: 29973808
DOI: 10.1100/tsw.2002.34
Article ID: 389146
Article version: 1
Subjects: Short Report

Beyond the Genome: A Drug for Every Gene?

Bailey David S. *
De Novo Pharmaceuticals, 59 St Andrews Street, Cambridge, CB2 3DD, UK
*David S. Bailey: david.bailey@denovopharma.com
Publication date: 2002
Electronic publication date: 2002 May 16
Volume: 2
First page: 68
Last page: 72
Copyright: Copyright © 2002 David S. Bailey.
Copyright year: 2002
License: This is an open access article distributed under the Creative Commons Attribution License, which permits unrestricted use, distribution, and reproduction in any medium, provided the original work is properly cited.
License URL: https://creativecommons.org/licenses/by/3.0/

==============================
Advances in Gene Technology: The Genome and Beyond –
Structural Biology for Medicine (Proceedings of the 2002 Miami
*Nature Biotechnology* Winter Symposium)
*TheScientificWorld* 2002, 2(S2), 68–72
ISSN 1532-2246; DOI 10.1100/tsw.2002.34

### BEYOND THE GENOME: A DRUG FOR EVERY GENE?

David S. Bailey
De Novo Pharmaceuticals, 59 St Andrews Street, Cambridge, CB2 3DD, U.K.
david.bailey@denovopharma.com

Genomics is revolutionizing the way in which we think about biology. Downstream technologies such as proteomics and structural biology now underpin our understanding of biological structure and function, while genome-wide transcriptome and proteome analysis allow us to gain an impression of the dynamic processes underlying biochemistry and physiology. Nowhere is the impact of genomics greater than in drug discovery. Genomics is now influencing the evolution of the pharmaceutical industry.

This presentation will focus on emerging technologies in genomics-based drug design and chemoinformatics as solutions for new lead discovery, the starting point of drug development. It will highlight some of the opportunities and challenges in industrializing the drug design process, and will address the emerging bottlenecks in reducing the design process to genome-wide application.

### The Importance of Genome-Wide Informatics in Target Discovery

The development of computational bioinformatics has been crucial to our understanding of the inter-relationships between specific therapeutic targets and the functional gene families to which they belong. The most comprehensive example of this has undoubtedly been the task of sequencing and assembling the human genome[1,2], a feat that lay at the very edge of our ability to handle such complex computational tasks. Assigning function to the individual genes within the genome is a continuing challenge.

The information produced from both public and private genome programs already provides a powerful foundation for future exploitation of genes as targets in drug discovery[3] and comprehensive mining of the human genome for therapeutic targets has uncovered a wealth of new opportunities for exploitation in molecular pharmacology and mechanism-based drug discovery[4].

### Traditional High-Throughput Screening Approaches for Lead Discovery

Until recently, the contribution made by genomics to drug discovery centred primarily on the production of protein targets for biochemical screening. With bioinformatics allowing the definition of specific therapeutically relevant genes and gene families, many targets have been reduced to discovery practice through high-throughput screening approaches.

However, the success of such random screening approaches against novel targets has been inconsistent. If the objective is to find at least one functional small molecule ligand for every protein, and the human proteome contains at least 50,000 proteins, then the scale of the task becomes immediately apparent. It is widely acknowledged in the pharmaceutical industry that high throughput screening (HTS) has not met the early expectations that many leads would be found rapidly[5].

There are sound theoretical reasons why hopes for HTS have not been born out:

- Chemical space for drug-like molecules is vast: greater than $10^{60}$ drug-like compounds could exist in principle[6].
- The number of different structures made to date is only $10^{7}$–$10^{8}$ and most of the molecules are based on conserved parental structures.
- The amount of structural diversity available is limited.
- Ligand binding is often sterically very specific. Tight binding pockets impose severe restrictions on the size of molecular fragments that can be fitted within them.

In-house compound collections commonly range from 50,000 to 1,000,000 different compounds. Clearly this number is minuscule compared with the potential size of the set of drug-like molecules.

### Moving Screening *in silico*: Virtual Screening Approaches

One way of increasing chemical diversity is through *in silico* screening of large libraries (real and virtual). Virtual screening methods attempt to overcome some of the deficiencies in the HTS procedure by dramatically increasing the size of the screening set. This extension also enables large numbers of virtual molecules (existing only *in silico*) to be screened as well. Virtual screening also offers large financial savings by cutting down the number of compounds to be screened experimentally since only those compounds that show acceptable docking results need be screened in the laboratory.

Docking algorithms require a set of coordinates that may be derived from crystallographic studies, NMR, or homology models of the binding site. Three docking approaches are possible: rigid docking, flexible ligand docking, and a combination of flexible site with flexible ligand docking[7]. Flexible ligand docking is currently feasible for million compound libraries.

Docking algorithms are highly dependent on the scoring function, a statistical measure of the interaction energy between the ligand and the site. These parameters are derived from ligand cocrystal data and binding measurements. A universal scoring function is the “holy grail” of automated computational drug design.

### The Contribution of Structural Biology

Thus, NMR- and X-ray-based structural biology approaches are now providing crucial information in the drug discovery process. This ability to visualize and exploit the detailed architecture of specific active sites has already led to major advances in drug discovery for particular targets[5], including HIV-protease. It is these early successes in structure-based design (SBD) that herald the advent of industrialization of the design process[6].

However, X-ray crystallography is a slow process compared with gene sequencing, and NMR is limited by the size of protein that can be examined effectively. The only efficient way to process sequence data is by large-scale homology modeling[8]. This procedure provides clues to the function of the proteins by identifying the folds and 3D motifs known to bind to specific ligands. Steady progress is being made, with perhaps 3D-computational models covering half the human proteome becoming available by 2003[9]. However, the direct applicability of such models to design has yet to be demonstrated on a large scale.

**The Complementarity of Ligand-Based Approaches**

Another key, complementary approach is ligand-based design (LBD). The way in which both biological and synthetic ligands associate with sites underpins their biochemical activity. In this context, the pharmacophore hypothesis, by reducing complex molecular and spatial information from a set of active molecules to a defined number of points, has been of great value for drug design and development.

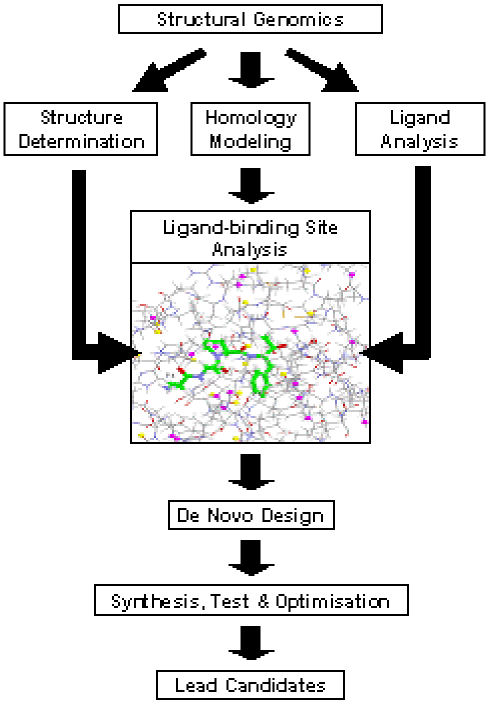

Where the structure of the site is unknown but a number of active compounds for the site are available, it is possible to use molecular similarity computations to infer the structure of the site. This does not yield a complete picture of the site but identifies key hydrogen-bonding site points and lipophilic regions. An algorithm, SLATE, has been written[10] to optimise the match between partially similar flexible molecules. Optimum superpositions can be obtained for the site points projected away from the ligand surfaces. These site points provide a minimal map of the putative receptor site. Drug design can then be carried out on these "supersurfaces" in a way that is analogous to site-directed design.

A worked example of the use of SLATE for ligand-based design has been published for the design of novel histamine H3 antagonists using information on the ligand alone without any information on the receptor[11].

**The Impact of *de novo* Methods of Design**

One of the most challenging aspects of drug design resides in the massive chemical space available for design, comprising $10^{60}$ theoretical drug-like compounds. One way of reducing the size of this space to manageable numbers is by applying combinatorial optimization techniques, to design within a 3D, biologically constrained site. The automation of this process is the basis of the proprietary *de novo* design approaches used at De Novo Pharmaceuticals.

The scoring functions for drug design and docking show many similarities, the aim being to prioritise a list of structures that can be compared for further assessment as candidates for synthesis. The addition of *de novo* methods of design to more traditional HTS and virtual screening approaches extends the former technologies into new areas of chemistry-led discovery and lead optimization[12].

**Chemoinformatics Architecture**

One further element is required if we are to capture the full benefit of these new approaches to drug design: an improved chemoinformatics infrastructure. The speed with which 3D chemical information is expanding is prodigious, bringing with it a need for a computational architecture capable of handling the increasingly large volumes of data, both biological and chemical, and querying it efficiently. The advent of large-scale data warehousing will necessitate the development of new data-mining tools.

The shape of future discovery campaigns may resemble the process outlined in the flow diagram below (taken from [5]). The challenge before us now is to exploit these emerging processes on a genomic scale.

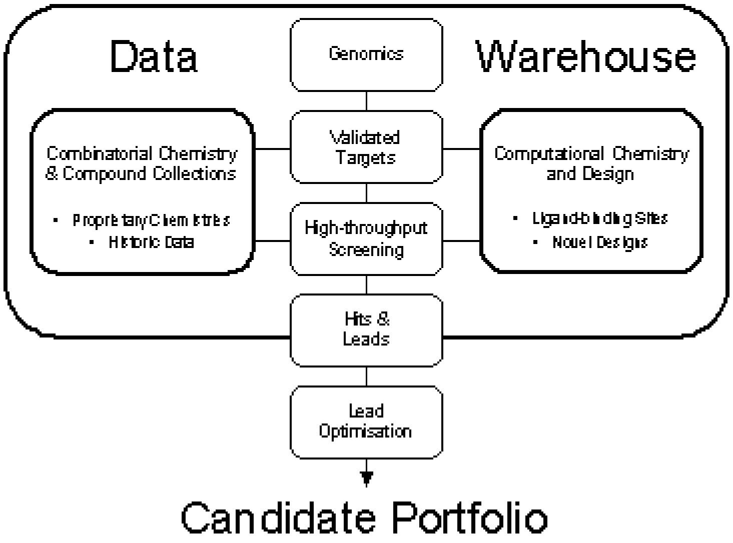

### REFERENCES

1. International Human Genome Sequencing Consortium (2001) Nature 409, 860–921.
2. Venter, J.C. et al (2001) Science 291, 1304–1351.
3. Bailey, D.S., Zanders, E., and Dean, P. (2001) Nat. Biotechnol. 19, 207–209.
4. Ohlstein E.H., Ruffolo, R.R., and Elliott J.D. (2000) Annu. Rev. Pharmacol. Toxicol. 40, 177–191.
5. Bailey, D. and Brown, D. (2001) Drug Discovery Today 6, 57–59.
6. Dean, P.M., Zanders, E., and Bailey, D.S. (2001) Trends Biotechnol. 19, 288–292.
7. Klebe G, Ed. (2000) Kluwer/ESCOM.
8. Sanchez, R. and Sali, A. (1998) Proc. Natl. Acad. Sci. U. S. A. 95, 13597–13602.
9. Sanchez, R., Pieper, U., Melo, F., Eswar, N., Marti-Renom, M.A., Madhusudhan, M.S., Mirkovic, N., and Sali, A. (2000) Nat. Struct. Biol. 7 (Suppl.), 986–990.
10. Mills, J.E.J., De Esch, I.J.P., Perkins, T.D.J., and Dean, P.M. (2001) J. Comput. Aided Mol. Design 15, 81–96.
11. De Esch, I.J.P., Mills, J.E.J., Perkins, T.D.J., Romeo, G., Hoffmann, M., Wieland, K., Leurs, R., Menge, W.M.P.B., Nederkoorn, P.H.J., Dean, P.M., and Timmerman, H. (2001) J. Med. Chem. 44, 1666–1674.
12. Bailey, D.S., Zanders, E., and Dean, P. (2001) Pharmacogenom. J. 1, 38–47.
